# The use of avulsed soft tissue for immediate full-thickness skin grafting in traumatic extremity injuries: A multi-institutional 10-year retrospective review of outcomes with technical considerations

**DOI:** 10.1016/j.jpra.2026.09.015

**Published:** 2026-09-16

**Authors:** Devin J. Clegg, Stephanie M. Mueller, Amy Du, LaYow C. Yu, Kyle R. Eberlin, Matthew J. Carty, Simon G. Talbot, Dennis P. Orgill

**Affiliations:** a Mass General Brigham, Department of Surgery, Division of Plastic and Reconstructive Surgery, Boston, MA, USA; b Harvard Medical School, Boston, MA, USA

**Keywords:** Full-thickness skin graft, Avulsion, Extremity, Trauma, Reconstruction

## Abstract

**Background:**

Avulsion or degloving injuries of the extremities are often caused by high-energy trauma and can be associated with high morbidity. Multiple techniques have been proposed for management, including immediate full-thickness skin grafting (IFTSG). Despite previous publication of our experience with this technique in 1999, awareness and implementation by surgeons remains poor. Outcomes of patients who underwent IFTSG from traumatically avulsed extremity soft tissues over a 10-year period were reviewed, with technical considerations included.

**Methods:**

A retrospective cohort study of patients with degloving/avulsion-type traumatic injuries of the extremities who underwent IFTSG from January 2015 to December 2025 at two academic institutions was performed. Patients without follow-up to complete healing or final outcome were excluded. Descriptive statistical analysis was performed, with case photos and surgical technique description included.

**Results:**

Fourteen patients were included. Upper extremity injuries occurred in 86% of patients. Associated fractures occurred in 29% of patients, and 71% had initial exposure of critical underlying structures. Mean IFTSG size was 140 cm^2^. Mean percentage of initial IFTSG take was 88%. Complete healing was achieved in 100% of patients, with a mean time of 87 days. Two patients required additional skin grafting. No patients underwent flap procedures, placement of skin substitutes, or proximal amputations.

**Conclusions:**

In appropriate patients, IFTSG of avulsed soft tissue in traumatic extremity wounds can reliably achieve complete healing with low complication risk and no additional donor site morbidity. Comparative studies are needed, but these promising findings should increase awareness of this technique for surgeons treating similar wounds.

## Introduction

Avulsion or degloving soft tissue injuries are often caused by high-energy trauma and can be associated with high morbidity. These injuries often lead to the delamination of skin and subcutaneous tissue from underlying muscle, connective tissues, or bone, creating a partially attached flap of soft tissue with reduced perfusion and exposure of underlying structures. While the true incidence of these injuries is unknown, they commonly occur in the extremities and are often found in combination with other injuries such as long-bone fractures and damaged blood vessels, muscle, tendons, or nerves.^1^, ^2^, ^3^, ^4^ The mechanisms of injury vary, and include motor vehicle collisions, machine-related injuries, crush injuries, bite injuries, sharp lacerations, and falls, particularly in the elderly.^1^, ^2^, ^3^, ^4^ While many of these injuries are considered external degloving or avulsions with resulting open wounds, they can also occur internally.^5^^,^^6^ Shear forces and hematomas can cause the overlying skin and subcutaneous tissues to separate from the underlying structures, leading to poor soft tissue perfusion or necrosis.^5^^,^^6^

Without appropriate treatment, these injuries can lead to complications. These include significant soft tissue loss through secondary skin necrosis, exposure of underlying structures resulting in increased risk of fracture malunion or nonunion, infection, scar contracture deformity and dysfunction, amputation, and even death.^2^ Many techniques have been proposed for management of these soft tissue injuries, including primary laceration repair with or without removal of subcutaneous tissues,^2^^,^^7^ creation of split-thickness skin grafts from the avulsed skin,^2^^,^^8^^,^^9^ cryopreservation of the avulsed tissue for replantation,^2^^,^^10^^,^^11^ dermal regeneration template or skin substitute placement,^12^^,^^13^ and the creation of full-thickness skin grafts from the avulsed soft tissue for immediate grafting.^1^^,^^7^^,^^12^^,^^14^, ^15^, ^16^, ^17^, ^18^

It has long been recognized by many plastic surgeons that avulsed but partially attached soft tissues that are reapproximated have high risk of necrosis, but if removed, thinned, and replaced as a skin graft, have an increased chance of survival. Immediate full-thickness skin grafting (IFTSG) involves division of the remaining attachments of the avulsed flap and “defatting”, which is the process of removing subcutaneous adipose tissue retained within the avulsion flap. As subcutaneous tissue and fat is poorly vascularized and acts as a physical barrier to imbibition, inosculation, and capillary growth from the recipient site, its removal has been demonstrated to decrease the risk of skin graft necrosis.^19^, ^20^, ^21^ The remaining dermis and epidermis from the injured tissue may then be used as a full-thickness skin graft. This converts the avulsed tissues from a poorly perfused soft tissue flap to a full-thickness skin graft with lower metabolic demand, improved exposure of the dermal vascular plexus for apposition to the vascularized wound bed, and preservation of as much viable tissue surface area as possible. In 1999 we published our report of five patients with nine avulsion injuries describing this approach, which also included the use of negative pressure wound therapy (NPWT) as an adjunct to healing.^17^

Despite our previous publication and active involvement in trauma care at our institutions, it appears that many surgeons are not aware of this technique, with cases of massive soft tissue defects resulting from not using this readily available donor location. The current literature on IFTSG largely consists of individual case reports or small case series, with some published several years prior to more modern improvements in traumatic injury management and wound healing techniques.^1^^,^^7^^,^^12^^,^^14^, ^15^, ^16^, ^17^, ^18^ As such, the purpose of this study was to review the outcomes of patients who underwent IFTSG from traumatically avulsed or degloved soft tissues at two large-volume academic centers over a 10-year period, as well as to provide technical considerations and increase awareness.

## Patients and methods

A retrospective cohort study was performed including patients with degloving or avulsion-type traumatic injuries of the extremities who underwent immediate full-thickness skin grafting from January 2015 to December 2025 at two academic institutions. This study was approved by our Institutional Review Board (Protocol number 2025P003110) and performed in adherence with the STROBE guidelines. Patients were identified by ICD codes for open wounds of the upper and lower extremities (ICD 10:S41, 10:S51, 10:S61, 10:S71, 10:S81, 10:S91, including all sub-codes) and CPT codes for full-thickness skin grafts to the upper and lower extremities (CPT 15,240, 15,241, 1520,15,221, including all sub-codes) prior to undergoing individual chart review for inclusion or exclusion. Exclusion criteria consisted of ages <18 years old, delayed full-thickness skin grafting (not at time of index operation), full-thickness skin grafting from a donor site that was not the avulsed soft tissue, incomplete records, and lack of follow-up to complete healing or final outcomes.

Data were obtained by manual chart review of the electronic medical record including demographics, comorbidities, medical/surgical history, traumatic injury etiology and description, operative details, complications, and outcomes during follow-up, including time to complete healing. Body mass index (BMI) was categorized according to World Health Organization criteria as underweight (<18.5 kg/m²), normal weight (18.5–24.9 kg/m²), overweight (25.0–29.9 kg/m²), and obese (≥30.0 kg/m²). Three representative patient cases were included, with written consent obtained.

Statistical analyses included descriptive statistics (count with percentage, and mean with standard deviation and range) performed in Microsoft Excel (Version 2603).

## Operative technique

### Wound evaluation and preparation

Avulsion or degloving-type wounds are evaluated for concurrent injuries and exposed structures. The avulsed soft tissue flap is evaluated for irreversible ischemia or other abnormalities that may preclude its use as a skin graft. Adjuncts that can be used to assess the viability of the avulsed tissue and the wound bed include intra-operative fluorescein or indocyanine green angiography. Non-viable, overly traumatized, or heavily contaminated tissues are debrided. Concomitant procedures such as the repair of fractures, vasculature, muscle, tendon, or nerve, are performed if required. Exposed structures are covered with adjacent viable and well-vascularized tissue to create a healthy wound bed that is suitable for grafting, specifically when periosteum, paratenon, or perineurium are injured or missing, as is standard in all skin grafting procedures. Hemostasis is then obtained in the wound bed to decrease the risk of post-graft placement hematoma. Once this is achieved, immediate full-thickness skin grafting (IFTSG) utilizing the avulsed soft tissue may be performed. If the wound bed or avulsed soft tissues are not deemed suitable for IFTSG, debridement is completed and temporary wound bed coverage techniques are then applied such as negative pressure wound therapy (NPWT) or simple dressings as alternative reconstructive modalities are planned. While no longer performed routinely at our institutions due to the concern for possible increased risk of infection, the grafts obtained from the avulsed tissue could also be significantly thinned and stored at 4 °C for delayed grafting within one week.

### Full-thickness skin graft creation and placement

Fig. 1 demonstrates the steps of IFTSG creation and placement. First, any remaining attachments of the soft tissue avulsed flap divided. Alternatively, the remaining attachments of the skin can be left in place if it does not limit the eventual reach or coverage of the graft. Subcutaneous tissue is then completely removed from the dermal layer of the avulsed flap. Once the full-thickness skin graft has been prepared, it is secured to the underlying wound bed in standard fashion utilizing absorbable or non-absorbable sutures or staples with minimal tension, ensuring that the graft is well-apposed and contoured to the underlying vascularized tissue for maximum coverage. Intermittent fenestrations or pie-crusting can be performed in the IFTSG to allow for fluid egress, and to decrease the risk of graft failure secondary to seroma or hematoma. However, this technique may negatively impact cosmesis if too frequent. An overlying dressing or bolster is then placed to assist with graft apposition, or alternatively NPWT can be utilized. In near-circumferential or circumferential IFTSG, the continuous negative pressure suction can be decreased to -75 mmHg for avoidance of potential distal extremity perfusion compromise.

**Fig. 1:**
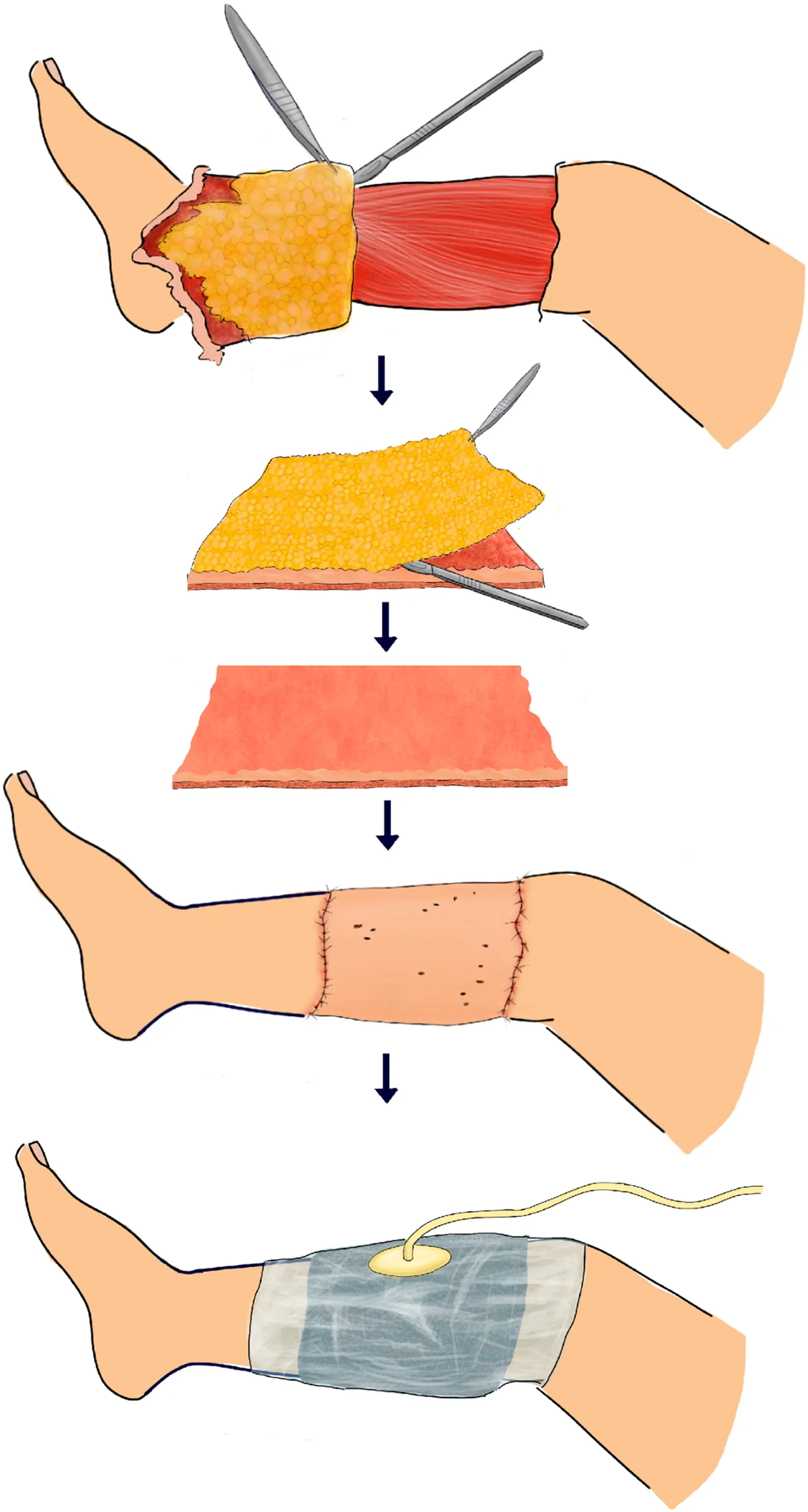
Surgical technique of immediate full-thickness skin grafting. This includes the division of remaining attachments of degloved soft tissue, removal of the residual subcutaneous tissue or “defatting”, resultant full-thickness skin graft, the securing of the immediate full-thickness skin graft to the underlying wound edges and bed with intermittent fenestrations performed to allow for fluid egress, and the application of negative pressure wound therapy as a graft bolster.

### Postoperative care

Postoperative care is similar to that of any full-thickness skin graft: immobilization of extremities to avoid shear forces, pressure avoidance, antibiotics if clinically indicated, and maintenance of a bolster dressing or NPWT for 5–14 days if appropriate by location. After initial dressings or bolster/NPWT removal and evaluation of graft take, daily dressing changes are performed to maintain moisture and protect the wound during continued healing.

## Results

Fourteen patients met criteria for inclusion in this study. Of these, 12 (86%) were male, and two were female (14%), with a mean age of 57 years (±20, 20–87). Thirteen (93%) patients identified as White. Patient comorbidities included obesity (*n* = 5, 36%), cardiovascular disease (*n* = 5, 36%), congestive heart failure (*n* = 3, 21%), peripheral vascular disease (*n* = 3, 21%), and immunosuppression (*n* = 2, 14%). The mean follow-up time of all patients was 240 days (± 290, 22–1000). Additional comorbidities are included in Table 1.

**Table 1:** Demographics and comorbidities.

Variable	Study population (*N* = 14)
Demographics	
Age *	57 (20), (20–87)
Sex	
Male	12 (86%)
Female	2 (14%)
Race	
Black	1 (7.0%)
White	13 (93%)
Comorbidities	
Body mass index	
Underweight (<18.5 kg/m^2^)	0 (0.0%)
Normal (18.5 – 24.9 kg/m^2^)	5 (36%)
Overweight (25.0 – 20.9 kg/m^2^)	4 (29%)
Obese (≥ 30.0 kg/m^2^)	5 (36%)
Cardiovascular disease	5 (36%)
Congestive heart failure	3 (21%)
Chronic obstructive pulmonary disease	2 (14%)
Diabetes	0 (0.0%)
Chronic kidney disease	2 (14%)
Peripheral vascular disease	3 (21%)
Malnutrition	1 (7.0%)
Immunosuppression	2 (14%)
Coagulopathy	1 (7.0%)
History of chronic wounds to injured site	0 (0.0%)
Prior radiation to injured site	0 (0.0%)

Notes.*^⁎^*Mean (SD), (range).

Avulsion or degloving injuries occurred to the upper extremity in 12 (86%) patients, and the lower extremity in two (14%) patients (Table 2). Most injuries were isolated (*n* = 12, 86%), while two (14%) patients had polytrauma. The mechanism of injury was blunt in seven patients (50%) and penetrating in seven patients (50%). Of the blunt injuries, four patients had suffered mechanical falls, with two patients developing hematomas of the lower extremity with resulting skin avulsion and skin threatening. Associated fractures occurred in four (29%) patients, with injuries resulting in initially exposed structures in ten (71%) patients.

**Table 2:** Injury details.

Variable		Study population (N = 14)
Location		
Upper extremity	12 (86%)	
Lower extremity	2 (14%)	
Classification		
Isolated injury	12 (86%)	
Polytrauma	2 (14%)	
Etiology		
Blunt	7 (50%)	
Mechanical fall	4	
Motor vehicle crash	1	
Crush injury	1	
Minor trauma	1	
Penetrating	7 (50%)	
Sharp laceration	2	
Saw injury	3	
Dog bite	1	
Gunshot wound	1	
Injury severity		
Associated fractures	4 (29%)	
Critical structures exposed	10 (71%)	
Tendon	8	
Bone	3	
Blood vessels	5	
Nerve	4	

Notes.n (%).

Operative details are contained in Table 3. The recipient site of the IFTSG was the forearm only in four (29%) patients, the forearm and hand in two (14%) patients, the hand only in two (14%) patients, and the distal lower extremity in two (14%) patients. The remainder included the digits. The mean IFTSG size was 140 cm^2^ (±290, 2–1100). NPWT was used in three (21%) patients at the time of grafting. Most (*n* = 10, 71%) patients had concomitant procedures performed at the time of IFTSG placement, such as fracture, tendon, muscle, blood vessel, or nerve repair. The mean number of procedures performed during the patient’s index hospitalization was one (±2, 1–8).

**Table 3:** Operative details.

Variable	Study population (*N* = 14)
IFTSG Recipient site	
Forearm	4 (29%)
Forearm, hand	2 (14%)
Hand	2 (14%)
Thumb	1 (7.0%)
Small finger	1 (7.0%)
Ring finger	1 (7.0%)
Forearm, hand, ring finger, small finger	1 (7.0%)
Distal lower extremity	2 (14%)
IFTSG size (cm^2^)*	140 (290), (2–1100)
NPWT	3 (21%)
Concomitant procedures^±^	10 (71%)
Number of procedures*	1 (2), (1–8)

Notes.*Mean (SD), (range).

IFTSG, immediate full-thickness skin graft; NPWT, negative pressure wound therapy.

^±^Concomitant procedures occurred in 10 patients. They included muscle/tendon repair, nerve repair, neurolysis, vein repair, open reduction with internal fixation, open reduction with external fixation, carpal tunnel release, hematoma evacuation, and fasciotomy.

Outcomes are reported in Table 4. The mean percentage of IFTSG take at first dressing or bolster/NPWT removal was 88% (±23, 15–100). Complete healing was achieved in all 14 (100%) patients during follow-up, with a mean time to complete healing of 87 days (±56, 22–170). Partial graft necrosis occurred in two (14%) patients and infection in one (7%) patient. Four (29%) patients required partial debridement of the IFTSG, with a mean number of debridements performed of one (±2, 0–7). Two (14%) patients required additional skin grafting, both split-thickness skin grafts, with a mean size of 690 cm^2^ (±720, 180–1200). No patients underwent flap procedures, placement of skin substitutes, or proximal amputations.

**Table 4:** Outcomes.

Variable	Study population (*N* = 14)
Healing	
Percent IFTSG take^*,±^	88 (23), (15–100)
Complete healing achieved	14 (100%)
Time to complete healing (days)*	87 (56), (22–170)
Complications	
Partial IFTSG necrosis	2 (14%)
Infection	1 (7.0%)
Additional procedures performed	
Partial debridement of IFTSG	4 (29%)
Number of debridements*	1 (2), (0–7)
Additional skin grafts	2 (14%)
STSG	2
Size of addition STSG (cm^2^)*	690 (720), (180–1200)
Flaps	0 (0.0%)
Skin substitutes	0 (0.0%)
Proximal amputation	0 (0.0%)
Follow-up time (days)*	240 (290), (22–1000)

Notes.*Mean (SD), (range).

^±^IFTSG take was defined by percent take when dressings/bolster/wound vacuum were removed for the first time postoperatively.

IFTSG, immediate full-thickness skin graft; STSG, split-thickness skin graft.

To demonstrate the application of the IFTSG technique, patient case photos are included (Figs. 2–4) with additional patient details in Table 5. Fig. 2 demonstrates patient number 3 (Table 5), a 61-year-old male who presented with an avulsion injury of the dorsal forearm following a saw injury with initially exposed muscle, tendon, and nerve. He was treated with debridement, nerve repair, partial primary closure, and IFTSG from the avulsed soft tissue with healing 35 days postoperatively. Fig. 3 demonstrates patient number 4 (Table 5), a 70-year-old male who presented with an avulsion injury of the dorsal forearm and hand following a mechanical fall. In the emergency department he was treated with debridement, partial primary closure, and IFTSG from the avulsed soft tissue with healing achieved by 37 days. Fig. 4 demonstrates patient number 9 (Table 5), a 71-year-old female who presented with a large hematoma of the lower extremity following blunt trauma while on therapeutic anticoagulation resulting in threatened and avulsed soft tissues. She underwent hematoma evacuation, debridement, and a 300 cm^2^ IFTSG with NPWT placement. She subsequently required multiple debridements of the IFTSG with a comparably smaller additional 180 cm^2^ STSG. She ultimately achieved complete healing by postoperative day 120. These patient cases demonstrate a few of the various injury presentations for which the IFTSG technique can be applied successfully, leading to minimized or no additional donor site morbidity.

**Fig. 2:**
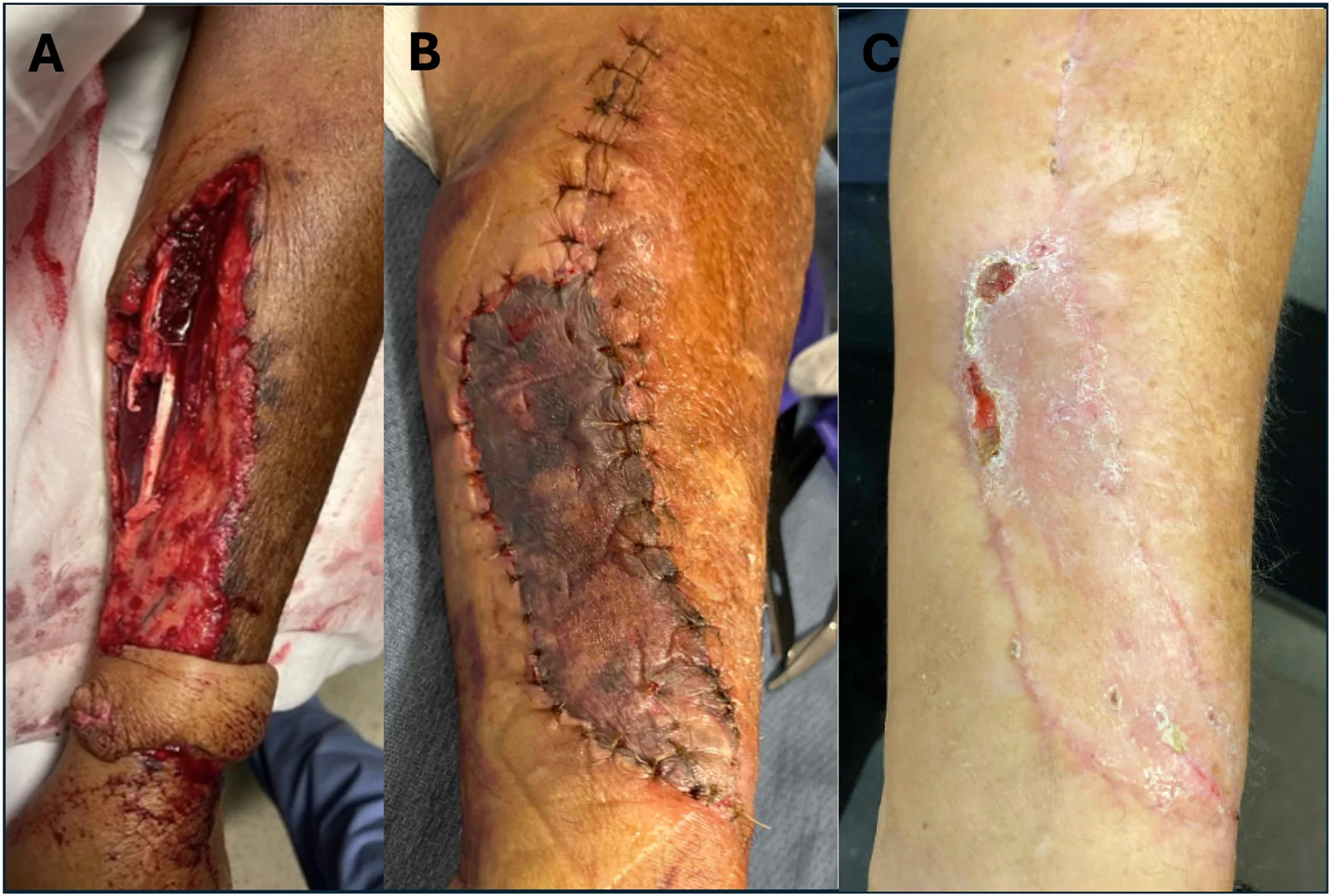
(A) 61-year-old male with an avulsion injury of the dorsal forearm following a saw injury, treated with debridement, nerve repair, partial primary closure, and IFTSG from the avulsed soft tissue. (B) Nine days postoperative evaluation. (C) 35 days postoperative evaluation.

**Table 5:** Patient operative information.

Patient number	IFTSG Recipient site	Size of IFTSG (cm^2^)	Concomitant procedures	NPWT	Number of procedures during index admission	Percentage of IFSTG take	Partial graft necrosis	Infection	Partial debridement of IFTSG	Number of debridements of IFTSG	Additional STSG	Size of STSG (cm^2^)	Time to complete healing (days)
1	Hand, wrist, ring finger, small finger	32	None	No	1	95%	No	No	No	0	No	-	31
2	Thumb	3	None	No	1	15%	Yes	No	Yes	1	No	-	98
3	Forearm	150	Nerve repair	No	1	95%	No	No	No	0	No	-	35
4	Forearm, hand	25	None	No	1	100%	No	No	No	0	No	-	37
5	Forearm, hand	1080	Open reduction external fixation	Yes	8	75%	Yes	Yes	Yes	7	Yes	1200	151
6	Forearm	4	Nerve repair, tendon repair	No	1	100%	No	No	No	0	No	-	65
7	Small finger	4	Open reduction external fixation	No	1	100%	No	No	No	0	No	-	47
8	Hand	2	Nerve repair, muscle repair	No	1	100%	No	No	No	0	No	-	112
9	Lower leg	300	None	Yes	2	75%	No	No	Yes	5	Yes	180	120
10	Ring finger	2	Open reduction internal fixation, tendon repair	No	1	100%	No	No	No	0	No	-	159
11	Forearm	18	Vein repair	No	1	100%	No	No	No	0	No	-	22
12	Forearm	30	Nerve neurolysis	No	1	100%	No	No	No	0	No	-	22
13	Hand	36	Nerve repair, muscle repair, tendon repair, carpal tunnel release	Yes	2	80%	No	No	Yes	1	No	-	145
14	Lower leg	225	Hematoma evacuation, fasciotomy	Yes	1	100%	No	No	No	0	No	-	169

Notes. All IFTSG achieved complete healing.

IFTSG take was defined by percent take when dressings/bolster/wound vacuum were removed for the first time postoperatively.

IFTSG, immediate full-thickness skin graft; NPWT, negative pressure wound therapy; STSG, split-thickness skin graft.

**Fig. 3:**
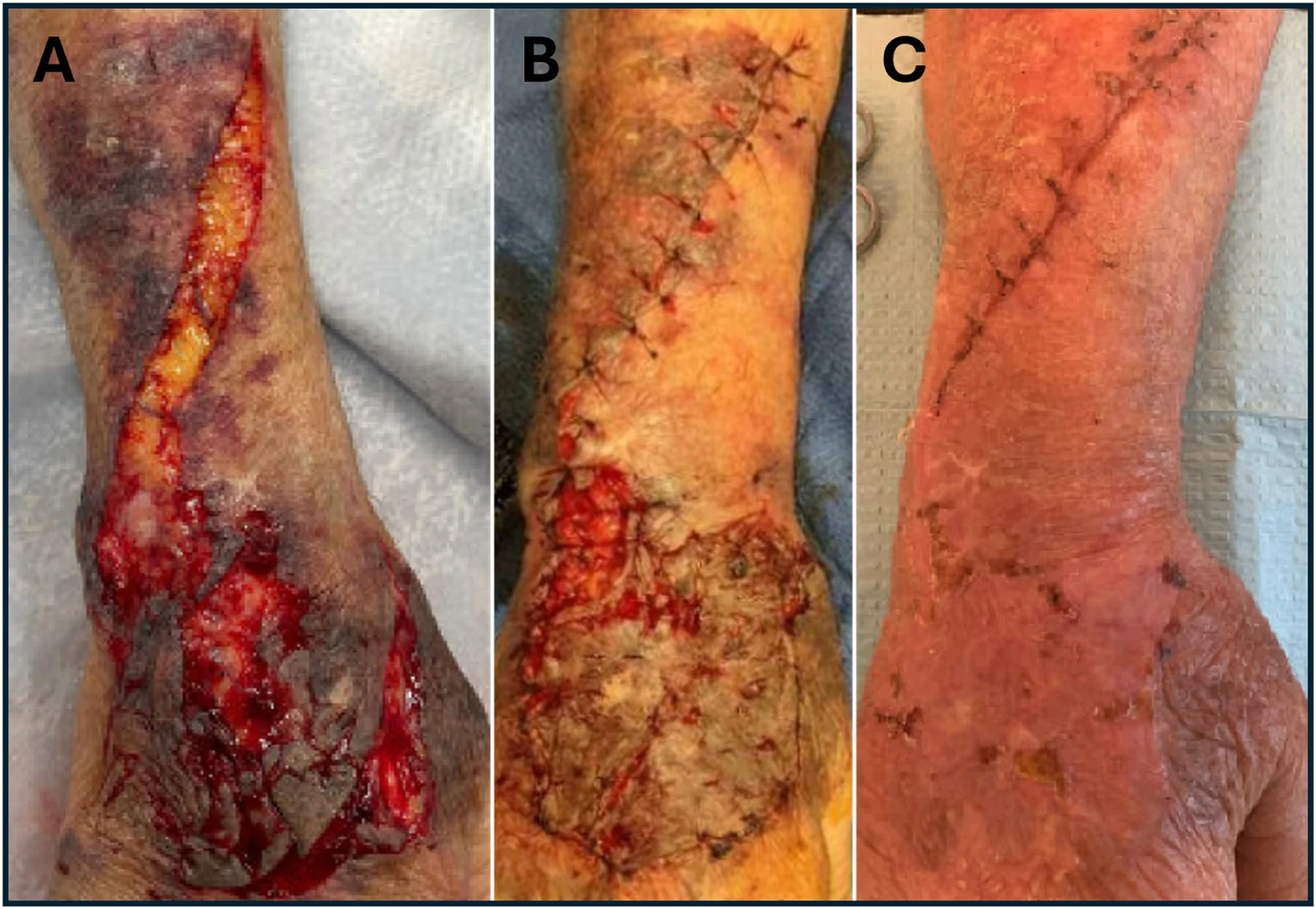
(A) 70-year-old male with an avulsion injury of the dorsal forearm and hand following a mechanical fall. (B) Treated with debridement, partial primary closure, and IFTSG from the avulsed soft tissue. (C) 37 days postoperative evaluation.

**Fig. 4:**
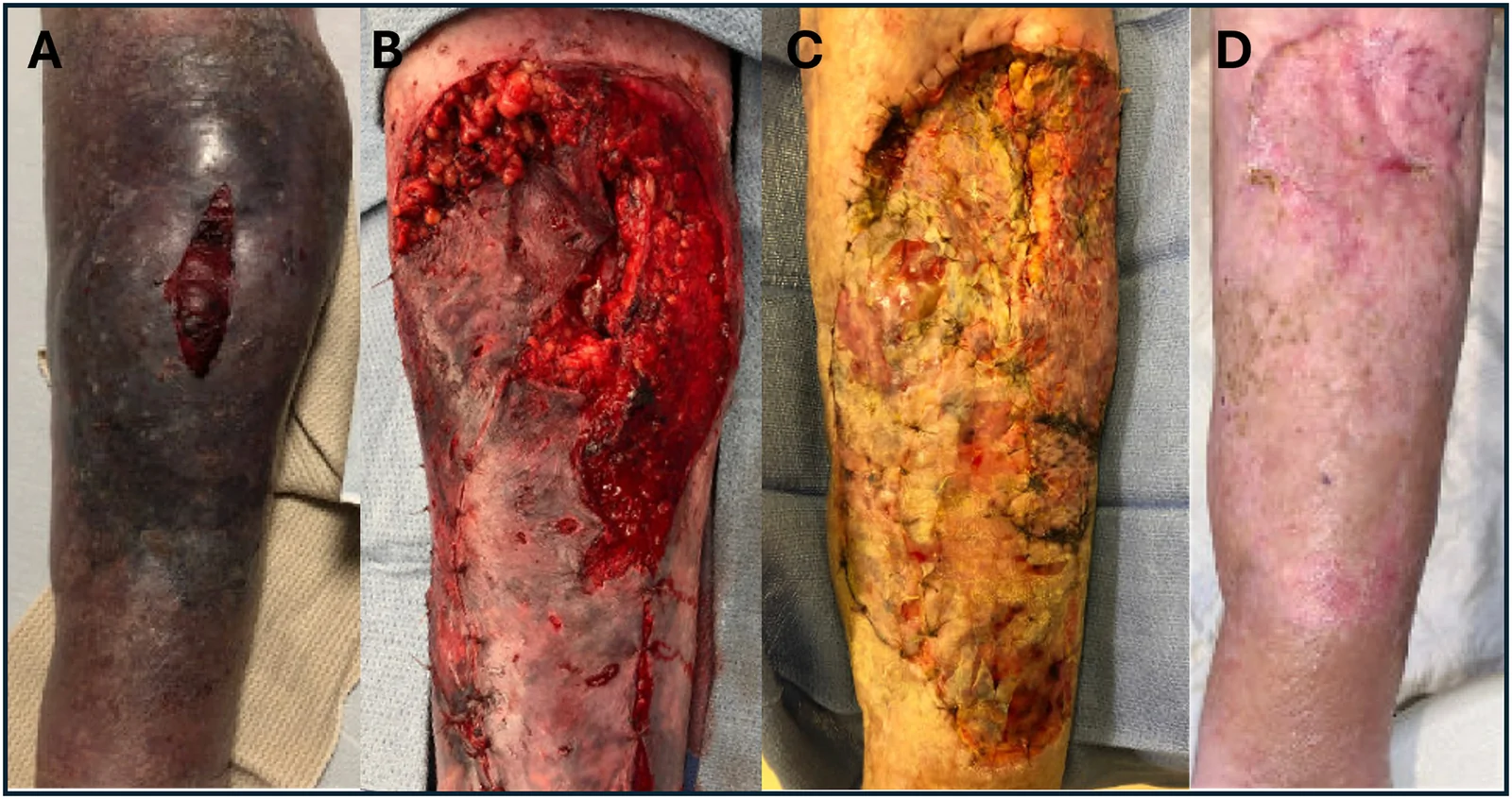
(A) 71-year-old female with a hematoma of the lower extremity following blunt trauma in the setting of anticoagulation. (B) Treated with hematoma evacuation, debridement, and IFTSG from the threatened and avulsed soft tissue, followed by additional STSG two-days postoperatively. (C) Ten days postoperative evaluation. (D) 120 days postoperative evaluation.

## Discussion

Overall, we found that all patients in this study achieved complete healing across a variety of traumatic mechanisms, comorbidities, extremity locations, and IFTSG sizes. The average time to complete healing was 87 days and ranged from 22 days to 170 days. The average IFTSG size was 140 cm^2^ and ranged from 2 cm^2^ to 1100 cm^2^. Complications included partial graft necrosis resulting in debridement occurring in two patients, and infection treated with oral antibiotics in one patient. In total, four patients underwent debridement of the IFTSG including two with partial graft necrosis, and two with incomplete graft take, with an average of one debridement performed amongst those patients. Additional skin grafts were required in two patients, and both were split-thickness skin grafts. No patients had complete IFTSG loss, required secondary reconstruction with flaps or skin substitutes, nor required a proximal amputation. As the IFTSG came from the patient’s own avulsed soft tissue, there was no donor site morbidity.

“Survive as a graft or die as a flap” is an adage that describes the pathophysiology that underlies the reasoning for utilizing this technique. Degloved or avulsed tissues are often perfused from small remaining attachments, and even with seemingly adequate arterial inflow, they can have significantly impaired venous outflow due to the traumatic disruption of the more delicate and thin-walled veins, leading to tissue compromise. Additionally, retained subcutaneous tissues are poorly vascularized, increase metabolic demands, and act as a physical barrier to imbibition, inosculation, and capillary growth from the underlying vascularized tissue bed. Thus, conversion to an IFTSG has been proposed to allow the tissue to overcome these wound healing obstacles.^1^^,^^14^, ^15^, ^16^, ^17^, ^18^, ^19^, ^20^, ^21^

While little comparative data exists, this technique relies on foundational plastic surgery principles.^22^ It allows for early wound coverage, avoiding the potential complications of debridement alone without immediate coverage, such as desiccation, wound breakdown, and infection.^23^ As this technique utilizes “spare parts” that otherwise may not survive or be discarded, there is no additional donor site morbidity.^24^ If the IFTSG does not completely take or has partial necrosis, this can be debrided and replaced with additional coverage including a split-thickness skin graft at a potentially smaller size than would have been required previously. As seen in our patient cohort, this technique may also obviate the need for more extensive secondary reconstructive procedures such as flaps or skin substitute placement, which can be costly and increase length of stay.^25^, ^26^, ^27^, ^28^, ^29^, ^30^ As grafting is performed during the same operation as debridement and concomitant structure repair, this technique does not require a mandatory second anesthetic or operation as with banking or storage of grafts. In certain patients with isolated injuries, IFTSG may decrease hospital length of stay, as some patients may be candidates for same day postoperative discharge and continued outpatient wound care.

NPWT has been reliably demonstrated to improve the outcomes of skin grafts with improved graft take and decreased complications.^17^^,^^31^^,^^32^ While only 21% of our patients received NPWT, this likely reflects the location of the IFTSG recipient sites, many of which were on the digits, an area less amenable to NPWT application. In our experience, NPWT is a safe and reliable alternative to traditional bolster dressings when the recipient site location is appropriate.^17^ As discussed in the operative technique section, for near-circumferential or circumferential IFTSG, the authors recommend decreasing the continuous negative pressure suction to -75 mmHg for avoidance of potential distal extremity perfusion compromise. This technique was applied in both patients with lower extremity IFTSG recipient sites, as well as two patients with hand or forearm IFTSG recipient sites.

Despite the prior literature demonstrating positive outcomes utilizing this technique, including from our institution in 1999,^17^ awareness and implementation of IFTSG has seemingly remained poor. In our initial published case series, five patients with nine degloving injuries of the upper and lower extremities treated with IFTSG and NPWT application were described. IFTSG sizes ranged from 40 to 1200 cm^2^, with a mean graft take of 81% after NPWT removal.^17^ Over the last 25 years, the authors have found this technique to be reliable, as reflected by the current study’s mean graft take of 88%. As shown by the relatively low number of patients included over our 10-year study period, the utilization of the IFTSG approach has not greatly improved. As initial management of these injuries is often performed by trauma or orthopedic surgeons, increased education and awareness, along with early involvement of plastic surgeons, may help reduce potential unnecessary patient morbidity.

This study has limitations including its retrospective nature, smaller sample size that precludes multivariable statistical analysis, and lack of comparator. Despite these limitations, this cohort is a modern representation of ten years of patients at two metropolitan academic institutions.

## Conclusions

This study demonstrates that immediate full-thickness skin grafting of degloved or avulsed soft tissue in traumatic extremity wounds can be utilized to achieve complete healing with few complications and no additional donor site morbidity in appropriate patients. This technique can be applied to both upper and lower extremity traumatic injuries of different etiologies, can be utilized in patients with various comorbidities, and can be used in patients that require concomitant repair of underlying injuries. Early involvement of plastic surgery is recommended. Comparative studies to alternative techniques are needed, but these promising findings should increase awareness of this technique for surgeons treating similar wounds.

## Funding statement

This research did not receive any specific grant from funding agencies in the public, commercial, or not-for-profit sectors.

## Ethical approval statement

This research did not require specific approval from an ethics committee but conforms to the World Medical Association Declaration of Helsinki (June 1964) and subsequent amendments. This study was approved by our Institutional Review Board (Protocol number 2025P003110) and performed in adherence with the STROBE guidelines.

## Declaration of competing interest

The authors have no financial disclosures or conflicts of interest related to this manuscript. KRE is a consultant for Axogen Inc., Checkpoint Surgical Inc., Integra Lifesciences Inc., Tissium, Tulavi Therapeutics Inc., and Biocircuit. SGT is a cofounder of Moral Injury of Healthcare, consultant for Six Therapeutics and Tissium, and a member of the Data Safety Monitoring Board of Neuraptive. DPO is a consultant for Smith and Nephew, Urgo Medical, MTF Biologics, and PERI.
